# New Insights into the RNA-Binding and E3 Ubiquitin Ligase Activities of Roquins

**DOI:** 10.1038/srep15660

**Published:** 2015-10-22

**Authors:** Qi Zhang, Lixin Fan, Feng Hou, Aiping Dong, Yun-Xing Wang, Yufeng Tong

**Affiliations:** 1 Structural Genomics Consortium, University of Toronto, Toronto, ON M5G 1L7, Canada; 2 Small-Angle X-ray Scattering Core Facility, Center for Cancer Research of the National Cancer Institute, Frederick National Laboratory for Cancer Research, Leidos Biomedical Research, Inc., Frederick, MD 21702, USA; 3 Protein-Nucleic Acid Interaction Section, Structural Biophysics Laboratory, National Cancer Institute at Frederick, National Institutes of Health, Frederick, MD 21702, USA; 4 Department of Pharmacology and Toxicology, University of Toronto, Toronto, ON M5G 1L7, Canada

## Abstract

Roquins are a family of highly conserved RNA-binding proteins that also contain a RING-type E3 ubiquitin ligase domain. They repress constitutive decay elements containing mRNAs and play a critical role in RNA homeostasis and immunological self-tolerance. Here we present the crystal structures of the RNA-binding region of Roquin paralog RC3H2 in both apo- and RNA-bound forms. The RNA-binding region has a bipartite architecture composed of ROQ and HEPN domains, and can bind to stem-loop and double-stranded RNAs simultaneously. The two domains undergo a large orientation change to accommodate RNA duplex binding. We profiled E2 ubiquitin-conjugating enzymes that pair with Roquins and found that RC3H1 and RC3H2 interact with two sets of overlapping but not identical E2 enzymes to drive the assembly of polyubiquitin chains of different linkages. Crystal structures, small-angle X-ray scattering, and E2 profiling revealed that while the two paralogs are highly homologous, RC3H2 and RC3H1 are different in their structures and functions. We also demonstrated that RNA duplex binding to RC3H2 cross-talks with its E3 ubiquitin ligase function using an *in vitro* auto-ubiquitination assay.

Post-transcriptional regulation of RNA by RNA-binding proteins (RBPs) is crucial in RNA homeostasis and gene translation. Aberrant expression, mutation, or malfunction of RBPs are associated with many human diseases, including neurological disorders, cancers, and autoimmune diseases^1^,^2^,^3^. Roquins are a family of RNA-binding proteins critically involved in maintaining peripheral immunological tolerance and preventing autoimmune diseases^4^. A *sanroque* mutation (Met199Arg) in the mouse Roquin gene *Rc3h1* leads to the production of high titre autoantibodies and a phenotype resembling human systemic lupus erythematosus (SLE)^5^. The human genome encodes two Roquin paralogs, RC3H1 and RC3H2. The N-terminal regions of these two proteins are highly homologous and are composed of a RING (Really Interesting New Gene)-type E3 ubiquitin ligase domain, followed by a unique, so-called ROQ domain of approximately 300 amino acid residues and a CCCH-type ZnF domain^5^. The mainly unstructured C-terminal half of each molecule is less conserved and is postulated to be involved in protein-protein interaction. The ROQ domain binds to a highly conserved, stem-loop-forming constitutive decay element (CDE), which is found in the 3′ UTR of many mRNAs encoding regulators of development and inflammation^6^. Roquins are localized to cytosolic stress granules through the binding of the ROQ domain with RNAs^7^ and to the processing bodies (P-bodies) through the interaction of the C-terminus with the protein components of RNA-decapping complexes^8^. Both stress granules and P-bodies are critical organelles that regulate mRNA translation and stability. Recently, RC3H1 has also been found to regulate microRNA homeostasis by directly binding with microRNAs and the RNA-induced silencing complex^9^.

Although RC3H1 and RC3H2 both repress the mRNAs of genes that control T follicular helper cell development, such as the inducible T-cell co-stimulator (ICOS), and the activation-induced tumor necrosis receptor superfamily 4 (TNFRS4)^10^,^11^, they are not functionally redundant. For example, *Rc3h1* RING-less and *Rc3h1*-null mice die prematurely, while *Rc3h2* RING-less mice are viable and healthy^11^, suggesting that the RING domain is important for the functional dissimilarity of the two paralogs.

Ubiquitination is the conjugation of a small regulatory protein, ubiquitin, to substrate proteins. This post-translational modification is catalyzed by a three-enzyme cascade (E1 activating enzyme, E2 conjugating enzyme, and E3 ligase) and plays an important role in almost all biological processes, such as proteasomal degradation, protein endocytosis and trafficking, DNA damage repair, and cell signaling^12^,^13^. E3 ubiquitin ligases can be classified into two main categories: RING-type and HECT (homologous with E6-associated protein C-terminus)-type, which differ in their three-dimensional structures and mechanisms of ubiquitin ligation. The RING-type E3 ubiquitin ligase domain interacts with both E2 enzymes and substrate proteins, assisting in the direct transfer of the activated ubiquitin from the E2 enzyme to the substrate. Currently, less than 20 of about 600 human E3 ubiquitin ligases have been shown to contain both a RING-domain and an RNA-binding domain^14^. It is becoming clear that these RNA-binding E3 ubiquitin ligases bring together two important cellular pathways involving ubiquitin signaling and RNA regulation. For example, the ubiquitination activity of the MEX-3C RING domain is required for the degradation of major histocompatibility complex (MHC) mRNA^15^; the E3 ubiquitin ligase/phosphatase complex MID1/PP2A binds to the expanded CAG repeat of huntingtin (HTT) mRNA and the ligase activity of MID1 regulates the translation of HTT mRNA^16^. However, it is unknown whether the ubiquitin ligase function of Roquins cross-talks with their RNA-binding function.

An imaging-based siRNA screen identified human RC3H2, but not RC3H1, as the E3 ligase that regulates the turnover of apoptosis signal-regulating kinase 1 (ASK1)^17^. *In vivo* ubiquitination assays, in both HeLa and HEK293 cells, confirmed that the RING domain of RC3H2 promotes the ubiquitin-proteasome-mediated degradation of ASK1 and regulates the cellular response to oxidative stress. The *C. elegans* ortholog of Roquin, RLE-1, similarly ubiquitinates the ASK1 ortholog NSY-1 and regulates pathogen infection in the worm^17^. RLE-1 was also found to induce the degradation of a FOXO family transcription factor, DAF-16, through the ubiquitin-proteasome pathway^18^. These studies attest that the RING domain of Roquin proteins is functional. Since proteasomal degradation is mediated by Lys48-linked polyubiquitination, it is not known whether Roquins are also involved in other ubiquitin signaling pathways mediated by non-Lys48 ubiquitin chains.

Here, we report the crystal structures of the human RC3H2 RNA-binding region in both apo- and intermediate early response 3 (Ier3) CDE RNA-bound forms. We discovered that RC3H2 can bind to stem-loop and double-stranded (ds) RNAs simultaneously. A comparison with RC3H1 structures suggests that the RNA-binding region has a bipartite architecture and that the orientation of the two subdomains is flexible to accommodate dsRNA binding. *In vitro* ubiquitination assay revealed that RC3H1 and RC3H2 can pair with two overlapping but dissimilar sets of E2 enzymes to drive auto-ubiquitination and form ubiquitin chains of different linkages. RNA binding influences RC3H2 ubiquitination activity, but has differential effects on polyubiquitination when RC3H2 is paired with various E2 enzymes. Thus, the cross-talk between RNA binding and ubiquitination by Roquins may be context dependent.

## Results

### The RC3H2 RNA-binding region has a bipartite domain architecture

**Figure 1:**
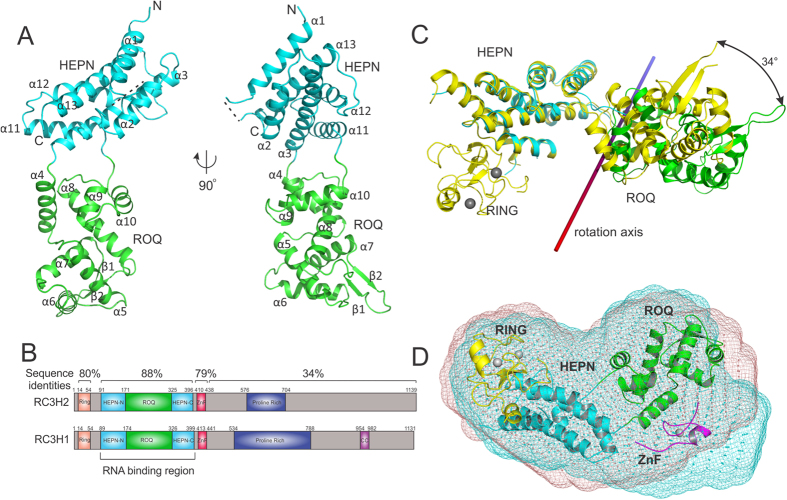
Characterization of the apo-form RC3H2 structure and comparison with RC3H1. (**A**) Ribbon diagram of the structure of the RC3H2 RNA-binding region. The ROQ and HEPN domains are coloured in green and cyan respectively. (**B**) Domain organization of full-length human RC3H1 and RC3H2. (**C**) Superposition of apo-RC3H2 (PDB:4Z30) and apo-RC3H1 structures (PDB:4TXA). The two structures are aligned in reference to the HEPN domain. The colour scheme for RC3H2 is the same as that in panel (**A**); RC3H1 is shown in yellow and zinc ions are shown as grey spheres. (**D**) SAXS envelope models of RC3H1 (a.a. 1–445, cyan) and RC3H2 (a.a. 1–442, red). For model fitting, the HEPN+ROQ domains of RC3H1 (PDB:4TXA) are replaced with the apo-structure of RC3H2, while keeping the orientation between the HEPN(cyan) and RING (yellow) domains unchanged. The ZnF domain (magenta) is built from PDB:1RGO. The structures were fitted to the SAXS envelope of RC3H2.

The ROQ domain of Roquin was initially identified based on sequence conservation among diverse species^5^ to be a region spanning a.a. 131–360, which is critical for its RNA-binding function^7^,^8^. Attempts to crystallize human RC3H1 (a.a. 1–445) yielded a crystal after four weeks. The crystal contained a polypeptide of approximately 17 kDa, much smaller than expected (Fig. S1A), which is likely a proteolytic product made by trace amounts of an unknown *E. coli* protease that survived purification. We then performed *in situ* proteolysis by adding 1:1,000 (w/w) elastase to crystallization drops^19^,^20^ and obtained a crystal diffracting to 1.7 Å. The solved crystal structure contains only a.a. 178–328 in the electron density map (PDB:4YWQ, Fig. S1B, Table 1) and the size matches the 17 kDa polypeptide of the original crystal. This suggests that a.a. 178–328 of RC3H1 forms a stable and independently folded domain, which conforms with the ROQ domain of RC3H1 that other groups have described^9^,^21^,^22^,^23^. Meanwhile, we designed several constructs of the RNA-binding region on the basis of secondary structure prediction^24^ and solved the crystal structure of a human RC3H2 fragment (a.a. 87–404). The structure of the RNA-binding region has an elongated shape consisting of 13 α-helices and one β-hairpin (Fig. 1A). The first three and the last three helices form a tightly packed unit that has little contact with the ROQ domain. This six-helix bundle was found to resemble an HEPN (higher eukaryotes and prokaryotes nucleotide-binding) domain based on a structural homologue search of RC3H1 using DALI^9^ (Fig. 1B). The HEPN domain of RC3H2 superimposes with that of RC3H1 with a root mean squared deviation (RMSD) of 0.52 Å for all atoms in 145 residues. An examination of the RC3H2 HEPN structure in the Protein Data Bank using PDBeFold^25^ retrieved several archaean proteins, the closest of which is a protein of unknown function (PDB:2Q00) from *Sulfolobus solfataricus*. The alignment Z-score is 5.2 and the RMSD value is 2.45 Å over the C_α_ atoms of 106 residues (Fig. S2).

The sequence identity between the RC3H1 and RC3H2 RNA-binding regions is very high (88%, Fig. 1B). However, an overlay of our RC3H2 ROQ structure with a recently reported RC3H1 structure^9^ containing RING, HEPN, ROQ, and CCCH-type ZnF domains (PDB:4TXA, Fig. 1C) revealed an unexpected orientation difference in the HEPN and ROQ domains of the RNA-binding region. Analysis using the Rapido algorithm^26^ confirmed that this region is composed of two rigid body moieties, which correspond to the HEPN and ROQ domains defined above. The two domains are connected through two flexible, proline-containing (Pro173 and Pro324) linkers and no buried interface exists between them. The overall RMSD of the RNA-binding region is 4.52 Å for all C_α_ atoms, while a flexible superposition that allows domain reorientation gives an RMSD_flex_ of 0.67 Å over 285 a.a. Superposition of the individual HEPN and ROQ domains of the two structures gives RMSDs of 0.42 Å and 0.52 Å, respectively, which suggests that these domains are compact and well folded. To compare the inter-domain orientation, we aligned the RC3H2 with the RC3H1 structure against the HEPN domain and found that the ROQ domain of RC3H2 is rotated 34° and shifted 2.5 Å away from the RC3H1 ROQ domain along an axis intersecting the two domains (Fig. 1C). It is unlikely that this difference is caused by the RING domain in the RC3H1 structure because the RING domain has a large contact area of 863 Å^2^ with the N-terminal helices of the HEPN domain, but makes no contact with the ROQ domain.

To determine whether the domain orientation difference between RC3H1 and RC3H2 is a crystallization packing artefact or a true representation of the dissimilarity between the two proteins, we further characterized the solution conformation of the RC3H1(a.a. 1–445) and RC3H2(a.a. 1–442) constructs containing the RING, HEPN, ROQ, and CCCH-type zinc finger (ZnF) domains using small-angle X-ray scattering (SAXS). Both proteins are monomeric in the solution and adopt an elongated conformation, with radii of gyration of 29.9 Å and 29.3 Å respectively, and a maximum dimension of 98.0 Å. The pair-distance distribution function of both proteins showed a tailing shape with two shoulder peaks (Fig. S3), suggesting a bipartite architecture. A calculation of the three-dimensional envelopes of RC3H1 and RC3H2 (Fig. 1D) revealed that, surprisingly, their shapes in the solution do not coincide. RC3H2 has more curvature between the two lobes than RC3H1. This is consistent with the observation in the crystal structures that the relative orientation of the HEPN and ROQ domains is different in the two proteins. We fitted the RC3H2 SAXS envelope using a model built from the apo-RC3H1 crystal structure (PDB:4TXA) by replacing the HEPN and ROQ domains of RC3H1 with those of RC3H2 while keeping the RING domain, and a homologous model built from PDB:1RGO to represent the ZnF domain of RC3H2 (36% sequence identity). We found the ZnF domain occupies a region close to the ROQ domain, although the orientation of the ZnF is speculative (Fig. 1D). Taken together, we postulate that in the apo-form, the HEPN and ROQ domains move relatively independently, and that the RING domain moves together with the HEPN domain, while the ZnF domain probably moves together with the ROQ domain in the solution.

### RC3H2 can bind to stem-loop and duplex RNAs simultaneously

**Figure 2:**
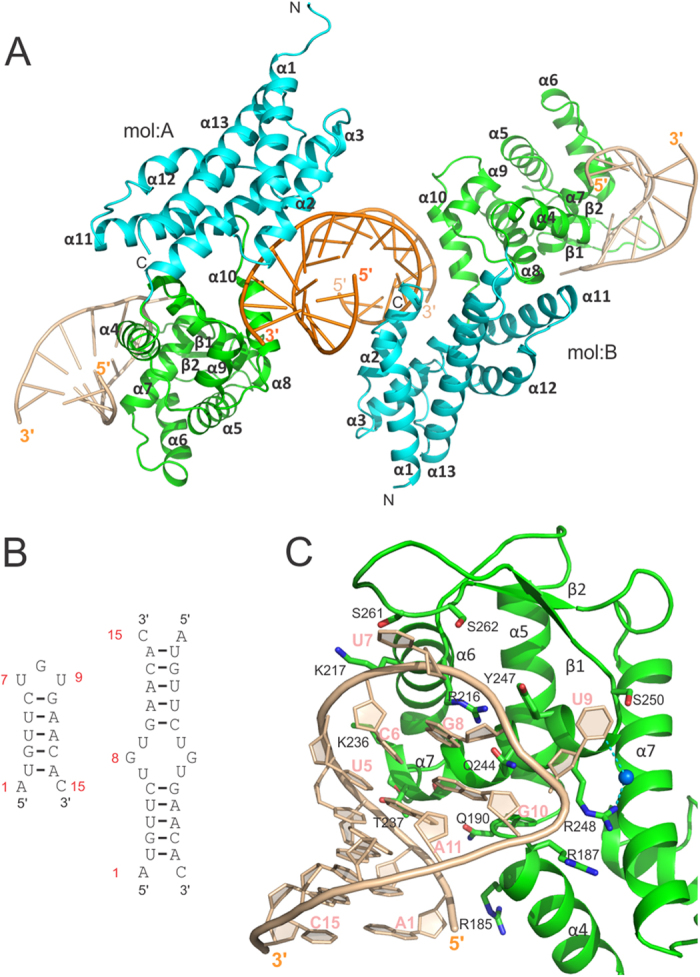
Interaction of RC3H2 with RNAs. (**A**) Ribbon diagram of the complex structure of RC3H2 and 15-nt Ier3 RNA in an ASU. (**B**) Schematic drawing of the RNA molecules shown in the structure. The numbering scheme of the nucleotides is also shown. (**C**) Interaction details of the stem-loop form Ier3 RNA and the ROQ domain. A water molecule bridging the pyrimidine base of U9 and the guanidinium group of Arg248 is shown as a blue sphere.

We next determined the crystal structure of RC3H2 in complex with a 15-nt constitutive decay element (CDE) of human intermediate early response 3 (Ier3) RNA at 2.5 Å resolution (Table 1). Surprisingly, each asymmetric unit (ASU) contains two protein molecules, two single-stranded RNAs, and one double-stranded (ds) RNA (Fig. 2A). The single-stranded RNA binds to the winged helix-turn-helix (wHTH)-like region^23^ of the ROQ domain, similar to previously reported RC3H1 structures in complex with different CDE RNAs^21^,^22^,^23^, while the RNA duplex binds to the HEPN and ROQ domains simultaneously.

The single-stranded Ier3 RNA forms a stem-loop structure containing five Watson-Crick base pairs and a UGU trinucleotide loop (Fig. 2B). The 5′ terminal ribonucleotide A1 forms a wobble base pair with the 3′ end C15, and this pairing is stabilized in the crystal packing by a Pi-Pi stacking between the pyrimidine rings of the A1 residues from two symmetry-related stem-loop RNAs (Fig. S4A). All nucleotides in the stem-loop RNA are clearly visible in the electron density map (Fig. S4B). The stem-loop RNA interacts with the ROQ domain mainly using most of the phosphate groups in the 5′ arm and the trinucleotide loop, while the 3′ half of the stem does not form direct interaction with the protein. The pyrimidine bases of the two uracils (U7 and U9) in the trinucleotide loop are flipped out (Fig. 2C). While no direct interaction is observed between the U7 pyrimidine base with the protein, the ribose ring of U7 forms hydrogen bonds with Ser262. The pyrimidine base of U9, instead, interacts directly with the side chain of Ser250 and indirectly with Arg248 through a bridged water molecule. The backbone of the stem-loop RNA is fixated by a large network of interactions with the side chains from helices α4, α6, and α7, and the β-hairpin of the ROQ domain (Table S1). The purine base of G8 in the trinucleotide loop forms a Pi-Pi stacking with the neighbouring C6-G10 Watson-Crick base pair in the CDE stem, and a cation-Pi stacking on the other side with the guanidinium group of the Arg216 side chain located at the N-terminus of helix α6 (Fig. 2C).

**Figure 3:**
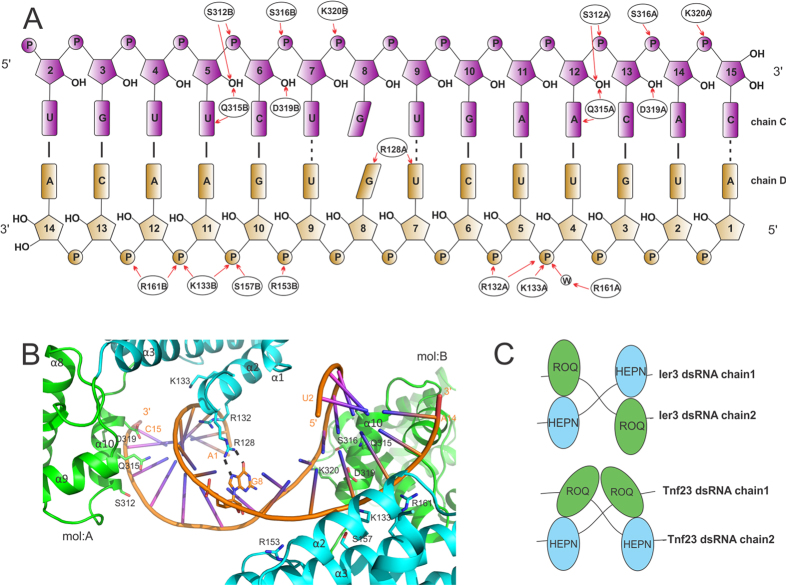
Interaction details of RC3H2 with double-stranded RNA. (**A**) Schematic drawing of RC3H2-Ier3 RNA duplex interactions. W stands for a water molecule. The suffix A or B in the amino acid annotation stands for the protein chains in an ASU. (**B**) Close-up view of the interactions between the side chain of Arg128 and the purine base of nucleotide G8. (**C**) Comparison of the different dsRNA-binding modes of RC3H2/Ier3 and RC3H1/Tnf23. Cartoon representation prepared by Q.Z.

Unexpectedly, our RC3H2/RNA complex structure contains an anti-parallel dsRNA. The RNA duplex has 10 Watson-Crick base pairs at the stem regions of the RNA molecules (Fig. 3A). All the nucleotides are clearly visible in the electron density map, except an adenosine (A1, chain C) and a cytidine (C15, chain D) at one end of the duplex (Fig. S4C). In the central trinucleotide UGU region, U7 from each RNA chain forms a wobble-type base pair with the U9 from the other chain. The purine rings of G8 from the two RNA chains are stacked. A detailed crystallographic analysis suggests that the two RC3H2 molecules in the ASU bind to the RNA duplex in a similar but not identical way (Fig. 3A). Both protein molecules use residues in the ROQ domain to interact with the backbone of one RNA strand (chain C), while residues in the HEPN domain interact with the backbone of the other RNA strand (chain D). Notably, side chains of Ser312, Gln315, and Asp319 of both protein molecules form hydrogen bonds with the hydroxyl group on the pentose ring of two adjacent ribonucleotides, suggesting an RNA- but not DNA-specific interaction. While the side chains of residue Gln315 in both protein molecules also interact with the nucleobases on the RNA duplex, the interaction does not seem to be nucleotide specific: one forms hydrogen bonds with the A12 purine ring and the other with the U5 pyrimidine ring. The most distinctive feature is the interaction between the guanidinium group of the Arg128 side chain from the HEPN domain of protein molecule A with the purine base of the central G8 from RNA chain D (Fig. 3B). This seems to be the only nucleotide-specific interaction between RC3H2 and the RNA duplex. Such an interaction does not exist for the Arg128 of the other protein molecule (chain B) in the asymmetric unit (ASU), probably because a cytidine takes the place of a guanosine at the equivalent position. The lack of this interaction also explains the missing densities for the terminal adenosine A1 and cytidine C15 from the RNA duplex. In compensation, the side chains of Ser157 and Arg153 from helix α2 of the HEPN domain of RC3H2 molecule B interact with the backbone phosphate groups of the RNA chain D. These extra interactions cause the HEPN domain in molecule B to rotate 12° and shift 0.5 Å closer to the RNA duplex along an axis intersecting the ROQ and HEPN domains compared to that of molecule A in the ASU (Fig. S4D). A schematic drawing of the interaction mode between RC3H2 and Ier3 dsRNA compared to that between RC3H1 and Tnf23 dsRNA is shown in Fig 3C (see discussion below).

### Characterization of the Roquin/RNA interaction in the solution

In our crystal structure of the RC3H1 ROQ domain (PDB:4YWQ) and those solved by other groups (PDBs:4QI0, 4ULW, 3X1O)^9^,^22^,^23^, two copies of RC3H1 with a contact area of 797 Å^2^ were observed in the ASU (Fig. S1B). A similar interface was also observed in the crystal packing of RC3H1 (a.a. 1–445)^9^. It was suggested that Roquin dimerizes through the ROQ domain, which enables it to bind with various RNA forms^23^. However, neither our apo-RC3H2 structure nor the RC3H2/RNA complex structure contains a similar protein-protein interface in the crystal packing. Indeed, a SAXS analysis of the RC3H1 ROQ domain in the solution suggests the ROQ domain dimerization observed at high protein concentration is non-specific^22^. This is also consistent with our SAXS measurement showing that Roquin constructs containing RING, HEPN, ROQ, and ZnF domains are monomeric in the solution. Hence, the dimer interface observed in the RC3H1 ROQ domain crystal structures is not physiologically relevant.

**Figure 4:**
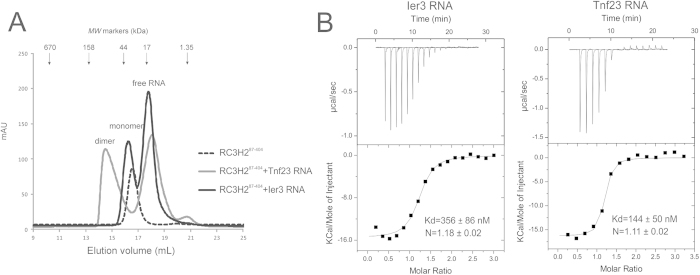
Characterization of the interaction of RC3H2 with RNAs in solution. (**A**) Size-exclusion chromatography of the RNA-binding region of RC3H2 in the absence and presence of Ier3 and Tnf23 RNAs. (**B**) Isothermal titration calorimetry measurement of the interactions between RC3H2 RNA-binding region with 15-nt Ier3 (left) and 23-nt Tnf RNAs (right). Upper panel, thermogram; lower panel, data fitted to one-binding site model.

We measured the oligomeric status of the RC3H2 ROQ domain in the presence and absence of four CDE RNAs^6^ (15-nt Ier3, 19-nt Roquin, 23-nt Tnf, and 27-nt Roquin2) in the solution using analytical size exclusion chromatography (SEC). The RC3H2 RNA-binding region is monomeric (apparent m.w. 32.0 kDa). The RC3H2/Ier3 complex exists predominantly as a 1:1 complex in the solution (36.8 kDa) instead of the 2:4 complex observed in the crystallographic ASU. In contrast, the RC3H2 /Tnf23 complex has a skewed elution profile with a maximum of approximately 85.5 kDa, making it almost twice the size of the RC3H2/Ier3 complex (Fig. 4A), while the peaks of the Roquin and Roquin2 CDE RNA complexes elute in between those of the RC3H2/Tnf23 and RC3H2/Ier3 complexes (Fig. S5A, S5B). Deduction of the accurate complex stoichiometry in solution by analytical SEC is hindered by two problems. Firstly, the apparent m.w. of RNA measured by SEC could be much larger than the expected m.w. when it is assumed to be a globular moiety^27^ and secondly, the ROQ domain has two RNA-binding sites and the RNAs could exist in either stem-loop or duplex forms. The elution peaks of various RC3H2/RNA complexes are broadly spanning a m.w. range of 37 kDa to 86 kDa when measured by SEC, suggesting that multiple forms of protein/RNA complex in different ratios may co-exist in equilibrium in the solution.

To confirm that the RNA-binding region of RC3H2 binds to dsRNA in the solution, we mutated three residues (Gln244Ala, Tyr247Ala, and Arg248Glu) that are involved in stem-loop RNA binding and measured their protein-RNA interactions using isothermal titration calorimetry (ITC). The wild-type RC3H2 binds to Ier3 RNA at a K_d_ of 356 ± 86 nM, and Tnf23 at 144 ± 50 nM (Fig. 4B). No measurable binding was observed between the triple-mutant and Ier3 RNA, while the mutant binds to Tnf23 with a K_d_ of 361 nM. We deduce that Ier3 RNA exists mainly in stem-loop form, while the Tnf23 RNA exists in both stem-loop and duplex forms in the solution. However, it cannot be ruled out that a small population of duplex form exists for Ier3 RNA, and that the crystallization process shifted the equilibrium between stem-loop and duplex RNA to create more RNA duplex/protein complex in the crystal lattice. We further tested the binding of RC3H2 with a 24-bp true dsRNA containing a segment of sequence from mouse inducible T-cell co-stimulator (ICOS) mRNA (XM_011251206), located 528 base downstream in the ICOS CDE sequence. The dsRNA was annealed from two complementary synthetic RNA chains, which do not form intra-chain base pairing. We discovered that the dsRNA can form a complex with RC3H2 (Fig. S5C), which confirmed that the RNA-binding region of RC3H2 actually binds to dsRNA.

Rapido analysis^26^ also identified Ser323 of RC3H2 (Thr326 in RC3H1) as the hinge residue connecting the ROQ and HEPN domains. Thr326 of RC3H1 (and possibly Ser323 of RC3H2) is a site subject to phosphorylation^28^, which may interfere with the flexibility of the linker region and alter the relative orientation of the interconnected domains. We introduced a phosphorylation mimic mutation, Ser323Glu, into the triple-mutant RC3H2 that does not bind to stem-loop RNA and examined its effect on dsRNA binding. The Ser323Glu mutation reduced the binding affinity between RC3H2 and Tnf23 RNA duplex from a K_d_ of 360 nM to 590 nM, as measured by ITC (Table S2). It also decreased the apparent m.w. of the RC3H2 and Tnf23 complex from 47.3 kDa to 39.9 kDa (Fig. S5D and S5E).

### Roquins are active E3 ubiquitin ligases

**Figure 5:**
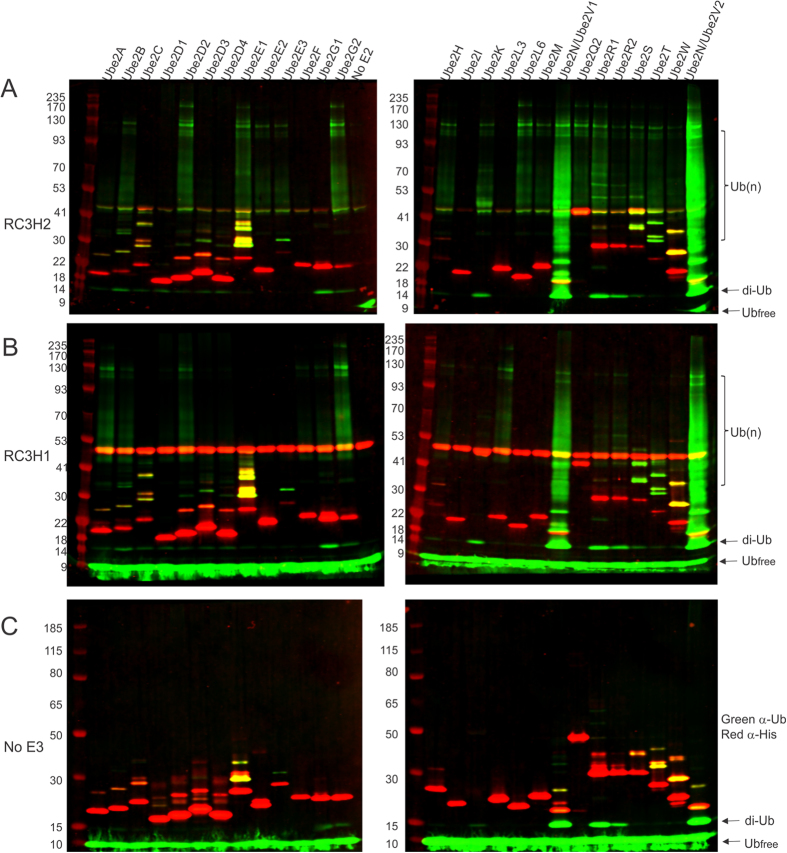
E2 ubiquitin conjugating enzyme profiling for Roquin E3 ubiquitin ligases. Ubiquitin (green) and His-tag (red) were visualized by immunoblotting after an auto-ubiquitination reaction catalyzed by RC3H2 (**A**), RC3H1 (**B**), or without E3 (**C**). The His-tag of the RC3H2 protein was removed, while that of RC3H1 was intact. All the E2 enzymes were His-tagged except UBE2K, which was GST-tagged. The E1 enzyme was not tagged. A set of control experiments for all the reaction systems without adding ubiquitin is shown in Fig. S9. Free ubiquitin, diubiquitin, and polyubiquitin chain bands are indicated by Ub_free_, di-Ub, and Ub(n), respectively. Free ubiquitin bands in panel (**A**) were mostly ran out of the gel. The free ubiquitin bands overlap with signals from the SDS-PAGE loading dye, so the intensity is not a reliable estimate of the amount. See main text for further discussion.

Although an *in vivo* ubiquitination assay has confirmed that RC3H2 is an active E3 ubiquitin ligase that regulates the proteasome-mediated degradation of human apoptosis signal-regulating kinase ASK-1^17^, it is unknown whether it is also involved in other ubiquitin signaling pathways, and whether RNA binding cross-talks with ubiquitination. We first carried out *in vitro* auto-ubiquitination assay using RING domain containing Roquin constructs (His6-tagged RC3H1 a.a. 1–445 and tag-removed RC3H2 a.a. 1–442) and found that both Roquins can interact with most of the E2 conjugating enzymes tested and drive polyubiquitination (Fig. 5A,B). However, we did not observe the auto-ubiquitination of RC3H1, as judged by the absence of any ubiquitinated RC3H1 bands, which should be stained both red and green at sizes larger than 53 kDa if they exist. This could be due to the lack of an appropriate lysine residue on RC3H1 as an auto-ubiquitination site. The observed polyubiquitin bands, as indicated by *Ub(n)* in Fig. 5A,B, could not have been generated by E3-independent E2 enzymes either, because no such polyubiquitin bands were observed for the majority of the E2s tested in the absence of RC3H1 or RC3H2 (Fig. 5C). In the few cases of UBE2R1, UBE2N/UBE2V1, and UBE2N/UBE2V2, where unanchored polyubiquitin chains were observed in the absence of E3 (Fig. 5C), addition of Roquins greatly increased the amount and length of the polyubiquitin chains formed (Fig. 5A,B). We also noticed that RC3H1 and RC3H2 show different preferences for the pairing E2 enzymes. For example, RC3H1 pairs with UBE2A, UBE2L3, UBE2F, and UBE2G1 and produces polyubiquitin chains, while RC3H2 does not; conversely, RC3H2 pairs with UBE2E2, UBE2E3, UBE2K, and UBE2Q2, while RC3H1 does not. On the other hand, RC3H1 and RC3H2 share a subset of E2 enzymes, e.g., UBE2B, UBE2D2, and UBE2G2, and drive polyubiquitination. Notably, Roquins show the strongest activity when paired with UBE2N/UBE2V1 or UBE2N/UBE2V2 E2 complexes and generate both short and long polyubiquitin chains (Fig. 5A,B). Using ubiquitin lysine mutants, we next examined the linkage of the polyubiquitin chains. RC3H2 drives formation of Lys63-specific polyubiquitin chains when paired with the UBE2N/UBE2V1 E2 complex, while it can use all seven lysines of the ubiquitin to form polyubiquitin chains when paired with UBE2D2 (Fig. S6A).

Since the RING domain of Roquins contacts the HEPN but not the ROQ domain, we postulated that dsRNA but not stem-loop RNA binding may cross-talk with ubiquitination. Selecting a few E2 enzymes, we tested the effect of Tnf23 RNA binding on the E3 ubiquitin ligase activity of Roquins. Tnf23 RNA did not significantly alter the auto-ubiquitination activity of RC3H2 when paired with the UBE2N/UBE2V1 E2 complex (data not shown). However, Tnf23 binding seemed to promote auto-ubiquitination driven by UBE2K, while inhibiting UBE2D2-mediated polyubiquitination (Fig. S6B).

When the complex structure of RC3H2 with Ier3 dsRNA is superimposed onto that of apo-RC3H1 (PDB:4TXA) in reference to the HEPN domain, we noticed a clash between the RING domain and the RNA duplex (Fig. S7A), suggesting that the RING domain must move to accommodate simultaneous binding of RC3H2 with dsRNA and E2 enzyme. We then tried to build a model of RC3H1/E2/RNA complex by superimposing the structures of RC3H1 (PDB:4TXA)^9^, RC3H1/Tnf23 duplex (PDB:4QIK)^21^ and the RNF8/UBE2N/UBE2V2 E2/E3 complex (PDB:4ORH)^29^. This superposition shows a clash between the E2 enzyme UBE2N with the second RC3H1 molecule in the structure (Fig. S7B). In either scenario, dsRNA binding is not compatible with E2 binding to the RING domain. While it is not straightforward to correlate RNA binding with the ubiquitination activity of Roquins, the close spatial distance between the RING domain and the RNA duplex and the clashes observed during modelling suggest there are some forms of cross-talk between HEPN/ROQ domain-mediated dsRNA binding and RING-domain-mediated ubiquitination.

## Discussion

### RNA binding and conformational change of Roquins

In this report, we solved the crystal structures of the RNA-binding region of RC3H2 in apo-form and in complex with Ier3 15-nt RNA in two topological forms. The RNA-binding region can be separated into two domains, ROQ and HEPN, which move relatively independently. This is consistent with several recent structural models of the paralog RC3H1^21^,^22^,^23^. It is noteworthy that while HEPN stands for higher eukaryotes and prokaryotes nucleotide-binding^9^ and the human SACSIN HEPN domain does interact with GTP^30^, we could not find any nucleotide binding motif in Roquin sequences, nor could we detect GTP binding using ITC (data not shown).

The winged helix-turn-helix motif of the ROQ domain^23^ is involved in the interaction with stem-loop RNA. Two stem-loop RNA-bound RC3H1 structures have been previously reported^21^,^22^. A comparison of our RC3H2 and 15-nt Ier3 RNA complex structure with the structure of RC3H1 in complex with the stem-loop form of 23-nt Tnf CDE RNA(PDB:4QI2)^22^ or 19-nt Hmg CDE RNA (PDB:4QIL)^21^ reveals that the C_α_ RMSDs of the ROQ domains are 0.39 Å and 0.28 Å, respectively, and that the key protein residues involved in the RNA interaction are identical for RC3H1 and RC3H2. All three RNAs use the 5′ arm backbone of a stem structure containing five Watson-Crick base pairs and a trinucleotide loop to interact with the ROQ domain. This suggests that stem-loop RNA recognition by the ROQ domain is sequence independent but conformation dependent, as has been noted previously^21^,^22^.

A Roquin/dsRNA complex structure was previously reported for RC3H1 and a 23-nt Tnf CDE RNA^21^ (Fig. S8A), which has a few noteworthy similarities and differences when compared to our RC3H2/Ier3 structure (Fig. 3A). In the RC3H1/Tnf23 structure, one of the central guanosine (G12) residue in the trinucleotide loop is not visible in the electron density map, while the purine ring of the other G12 forms a wobble-type base pairing with the U11 from the trinucleotide loop, creating a kink in the RNA duplex. In the RC3H2/Ier3 structure, electron density for all nucleotides in the central trinucleotide loop are clearly delineated (Fig. S4C). In the RC3H1/Tnf23 structure, the side chain of Arg131 forms hydrogen bonds with the purine base of an unpaired guanosine (G20 in one chain and G21 in the other) at the termini of the RNA duplex; while in the RC3H2/Ier3 structure, the side chain of the equivalent Arg128 interacts with the purine base of one central guanosine G8 (Fig. 3B) but not the guanosine in the other chain. Although the interaction between the protein and dsRNA mainly involves the RNA backbone and is topology dependent, the interaction between the side chain of an arginine and the purine base of a guanosine suggests there is weak sequence specificity of RNA recognition by Roquins. Topologically, the two RC3H1 molecules in the Tnf23 complex structure bind to the dsRNA in a head-to-head mode, and the two protein molecules contact each other through the ROQ domain. In contrast, the two RC3H2 molecules in the Ier3 complex bind to the dsRNA in a head-to-toe mode, and the two protein molecules do not interact (Fig. 3C). Briefly, the RC3H2/Ier3 complex has a pseudo-C2 rotation symmetry along an axis parallel to the RNA duplex, while the RC3H1/Tnf23 complex is symmetrical along an axis perpendicular to the RNA duplex.

The linker regions between the HEPN and ROQ domains are very flexible such that they can coordinate to “*clamp*” a dsRNA through domain movement. Indeed, when RC3H2 binds to the dsRNA, the ROQ domain rotates 42° along an axis intersecting the HEPN and ROQ domains to accommodate the interactions (Fig. S8B). The side chains of Glu333 and Arg340 from the HEPN domain form a strong interaction with the side chain of Arg294 and the carboxyl group of Ala291 from the ROQ domain (Fig. S8C), pulling the two domains closer. Similarly, when RC3H1 binds to dsRNA, the ROQ domain also undergoes a smaller rotation of 18° (Fig. S8D).

A putative phosphorylation site on the linker region (Thr326 in RC3H1 and Ser323 in RC3H2) may regulate the interaction of the HEPN/ROQ domains with dsRNA. The identity of the kinase that phosphorylates the S/T residue and how the phosphorylation affects RNA binding *in vivo* await further investigation. The interaction of the ROQ domain with dsRNA is mostly sequence independent, except for that of the side chain of Arg128 (of RC3H2) with the purine ring of a guanosine. The side chains (Ser312, Gln315, and Asp319 in Fig. 3A) from the HEPN domain also interact with the hydroxyl group on the pentose ring of two adjacent ribonucleotides. These interactions may be important for Roquins to bind dsRNA but not dsDNA.

Our structure corroborates the conclusion of a previous study that the dimerization of Roquins is not required for binding RNAs^23^. It also confirms that the two RNA-binding sites of the ROQ domain can bind to RNA simultaneously^21^. The 3D envelop model based on SAXS data suggests the CCCH-type ZnF domain packs against the ROQ domain and is close to the stem-loop RNA-binding site and the site for *sanroque* mutation Met199Arg. We hypothesize that the ZnF and ROQ domains form an auto-inhibited state, and that binding of stem-loop RNA to the ROQ domain may expose the ZnF domain. Since the ZnF domain could also potentially bind to RNA, the release of an auto-inhibited state may enhance the interaction between Roquin and RNAs. A crystal structure of the ROQ domain with a longer RNA transcript should provide further insight on this interaction.

### Roquin-mediated ubiquitination and cross-talk with RNA binding

Ubiquitin can use one of its seven lysines or the N-terminal methionine to form polyubiquitin chains of different linkages. These chains have various three-dimensional structures, dynamics, and surface properties and have vastly divergent physiological functions in the cell^13^,^31^. For example, the best-known Lys48-linked chain tags substrate protein for proteasomal degradation, while the Lys63-linked chain plays a crucial role in NF-κB signaling, DNA damage repair, and receptor endocytosis. E2 conjugating enzymes have intrinsic preference for chain linkage, which is usually not changed by RING-type E3 ligases^32^. The RING E3 ubiquitin ligase domain of Roquins adopts a typical cross-braced double zinc-finger fold^9^. This domain is important for the localization of Roquins to the stress granules, but not to the P-bodies^11^, suggesting that it is also involved in generating non-Lys48 polyubiquitin chains and may direct Roquin recruitment to the stress granules. Our E2 profiling results confirmed that Roquins can pair with many different E2 enzymes. UBE2N (also known as UBC13) is one such enzyme that can specifically assemble Lys63-linked ubiquitin chains^31^ and plays a key role in immune receptor signaling^33^. Not surprisingly, both RC3H1 and RC3H2 show high activity when paired with UBE2N and promote Lys63-chain formation. It was, however, unexpected that given the high sequence identity between the RING domains of RC3H1 and RC3H2, the proteins have preference for certain E2s. UBE2L3 is an E2 enzyme associated with several autoimmune diseases^34^,^35^,^36^, including SLE, via ubiquitination of the NF-κB receptor. It is interesting that RC3H1 but not RC3H2 can pair with UBE2L3 and drive polyubiquitination. Given the similarity of the phenotype of *sanroque* mice with the symptoms of human SLE, it is tempting to postulate that the RC3H1-UBE2L3 axis may act in the pathogenesis of the disease.

RNAs often exist in complex with proteins. The translational repression of mRNAs involves not only mRNA degradation, but also protein degradation^37^,^38^,^39^. Cross-talk between ubiquitination and mRNA degradation with the direct involvement of an RNA-binding E3 ubiquitin ligase has been demonstrated for MEX-3C^15^. Our ubiquitination assay result in the presence of dsRNA also suggests there is cross-talk between ubiquitination and RNA binding. Surprisingly, whether RNA binding enhances or reduces ubiquitination is E2 dependent. UBE2K is an E2 enzyme that assembles Lys48 linkage chains specifically^40^. RNA duplex binding to RC3H2 increases auto-ubiquitination driven by this enzyme. RNA binding may thus promote the degradation of associated ribonucleoproteins. In contrast, UBE2D2 is a highly promiscuous E2 enzyme that can assemble all of ubiquitin linkages. RNA binding may interfere with the formation of certain chain types.

In summary, our crystal structures of the RNA binding region of RC3H2 and ubiquitination assays confirmed that although the paralogs share many similarities, their functions are not identical. Future investigation of ubiquitination and RNA binding *in vivo* may provide insights on how ubiquitination cross-talks with RNA homeostasis and reveal other functional roles of the Roquin family E3 ligases.

## Methods

### Cloning, expression and purification

DNAs encoding the RING, HEPN, ROQ, and ZnF domains of human RC3H1 (a.a. 1–445) and RC3H2 (a.a. 1–442) and the RNA-binding region of RC3H2 (a.a. 87–404) were subcloned into either pET28-MHL (EF456735, for TEV cleavable N-terminal His6-tag) or pNIC-CH (EF199843, for uncleavable C-terminal His6-tag) vectors using the InFusion™ cloning kit (ClonTech). Site-directed mutagenesis was carried out using the PrimerSTAR HS DNA polymerase (Takara) and was verified by DNA sequencing. All proteins were over-expressed in a phage-resistant *E. coli* strain BL21 (DE3) harbouring the pRARE2 plasmid (Novagen) using Terrific Broth cultured in LEX Bioreactors (Harbinger Biotech). Protein production was induced using 0.5 mM IPTG overnight at 18 °C. All proteins were first purified using nickel-NTA agarose resin (Qiagen), and the His6-tag was then removed by TEV protease where applicable. Uncleaved protein and TEV protease were removed by another pass through the nickel-NTA resin. The proteins were further purified using cation-exchange chromatography (SP Sepharose Fast Flow, GE Healthcare), followed by size-exclusion chromatography (HighLoad 16/60 Superdex 200, GE Healthcare). The purified proteins were concentrated to final concentrations of between 25–35 mg/mL using an Amicon ultracentrifugal filter (m.w. cutoff 10 kDa, Millipore), flash frozen in liquid nitrogen, and stored at −80 °C. RC3H1(a.a. 1–445)and RC3H2 (a.a. 1–442) were stored in a buffer containing 25 mM Bis-Tris, pH 6.5, 500 mM NaCl, 1 mM DTT and 20 μM ZnCl_2,_ while the RNA-binding domain of RC3H2 was stored in a buffer containing 25 mM HEPES, pH 7.5, 150 mM NaCl and 1 mM DTT. For selenomethionine labelling, the target protein was over-expressed in *E. coli* using a prepacked M9 SeMet growth media kit (Medicilon) as per the manufacturer's instructions. All protein concentrations were determined by measuring UV absorbance at 280 nm using NanoDrop 2000 (Thermo Scientific).

### RNA preparation

Four single-stranded RNA oligos, Ier3: AUGUUCUGUGAACAC, Tnf23: ACAUGUUUUCUGUGAAAACGGAG, Roquin: AUAACUUCUGUGAAGUUGC, and Roquin2: UUAAUAACUUCUGUGAAGUUGUUUAC, and one RNA duplex dsICOS: UCUUCAGUCUAGACAGUUCGCUU (and its complementary sequence) were chemically synthesized and purified by HPLC (>90% purity, Integrated DNA Technologies), and dissolved in a buffer containing 20 mM Tris-HCl, pH 8.0, 150 mM NaCl, to a final concentration of 2 mM. All RNA concentrations were measured using UV absorbance at 260 nm. The RNA oligo stock solutions were heated at 98 °C for 5 minutes, then slowly cooled to room temperature, aliquoted, and stored at –80 °C. Repeated freezing and thawing were avoided.

### Crystallization

All crystals were grown at 20 °C using either the sitting or hanging drop vaporization methods. For RC3H1, the His6-tag-removed RC3H1 (a.a. 1–445) protein at a 25 mg/mL concentration was first mixed with elastase from porcine pancreas (Sigma-Aldrich) at a ratio of 1:1000 (w/w), then immediately mixed with a reservoir solution containing 25% PEG-8000, 0.2 M NaCl, 0.1 M HEPES, pH 7.5, 5% ethyl glycol at a ratio of 1:1. Crystals of the apo-form RC3H2 RNA-binding region were grown in a sitting drop mixed from 2 μL protein solution (C-terminal His6-tagged, 29 mg/mL) with 1 μL well solution containing 3.2 M NaCl, 0.1 M sodium acetate, pH 4.6. For the RC3H2/Ier3 RNA complex, N-terminal His6-tag-removed RC3H2 protein was first diluted to 6.7 mg/ml, then mixed with Ier3 RNA solution (2 mM) at a molar ratio of 1:1.5 before being set in a hanging drop. The drop was mixed from 1.5 μL protein/RNA complex and 1.5 μL well solution consisting of 17% PEG-10K, 0.1 M Bis-Tris, pH 6.5, and 5% ethyl glycol. All crystals for data collection were cryo-protected via an initial immersion in the corresponding well solution plus 10–15% ethylene glycol, then an immersion in Paratone-N (Hampton Research), before being flash-frozen in liquid nitrogen.

### Data collection and structure determination, refinement and analysis

X-ray diffraction data for apo-form RC3H2 was collected at 100K on CMCF beamline 08ID-1 at Canadian Light Source. The RC3H1 and RC3H2/RNA data were collected on beamline 19ID at Advanced Photon Source (APS), Argonne National Laboratory. All datasets were processed using the HKL-3000 suite^41^. The RC3H1 data was collected at the Se absorption edge and the structure was solved by the SAD (single wavelength anomalous dispersion) method^42^ using the SOLVE/RESOLVE program^43^. Both RC3H2 structures were solved by molecular replacement using PHASER^44^, with PDB entry 4QIK as the search template. COOT^45^ was utilized for model building and visualization. We employed REFMAC^46^ and PHENIX^47^ for restrained refinement. The final structure was validated by MOLPROBITY^48^. All structure graphics were prepared using PyMOL software (Schrödinger, LLC). Domain rotation was calculated using UCSF Chimera^49^.

### Isothermal titration calorimetry (ITC)

All ITC measurements were carried out at 25 °C on a MicroCal ITC200 (GE Healthcare). All protein and RNA samples were diluted from stock solutions into the same buffer containing 20 mM Tris-HCl, pH 8.0, 150 mM NaCl, 1mM TCEP before measurement. For each titration, 15–18 injections were performed by injecting 2 μl 200–500 μM RNA into a sample well containing 0.25 mL of 15–30 μM proteins. The concentrations of the proteins and RNAs were determined by measuring UV absorbance at 280 nm and 260 nm, respectively. Binding isotherms were plotted, analyzed, and fitted to a one-site binding model using Origin software (MicroCal Inc.). The dissociation constant K_d_, entropy, enthalpy, and Gibbs free energy were also calculated during curve fitting.

### Auto-ubiquitination assay and E2 profiling

All auto-ubiquitination was carried out in a 20 μL reaction system containing 100 nM E1 enzyme UBE1, 2 μM individual E2 enzymes, 2 μM RC3H1 (a.a. 1–445) or RC3H2 (a.a. 1–442) protein and 50 μM ubiquitin. The reaction buffer contained 20 mM Tris-HCl, pH 7.6 (4 °C), 50 mM NaCl, 1 mM β-mercaptoethanol and 5 mM MgCl_2_. All the enzymes, and RNA where applicable, were premixed, and ubiquitination was initiated by adding ATP to a final concentration of 2 mM. An E2 Screening Kit (UBPbio) was used for E2 profiling, which contained 27 His6-tagged E2 enzymes or complexes and one GST-tagged UBE2K. After incubation at 37 °C for 2 hours, SDS-PAGE loading buffer was added to stop the reaction. The proteins were electrophoretically separated on NuPAGE Novex 4–12% Bis-Tris gels (ThermoFisher) in MOPS SDS running buffer. Ubiquitination products were probed using a P4D1 monoclonal ubiquitin antibody (BioLegend). E2 enzymes were probed using a DyLight 680 conjugated anti-His antibody (Life Technologies). IRDye® secondary antibodies (LI-COR) were used for indirect detection and the results were visualized on an Odyssey CLx infrared imaging system (LI-COR). For chain linkage specificity test, a ubiquitin linkage screen kit I (UBPBio) was used.

### Size exclusion chromatography (SEC)

For SEC experiments, samples of the wild-type and mutant RC3H2 ROQ domains were mixed with different RNAs at a 1:3 molarity ratio and passed through an analytical column (Superdex 200 10/300, GE Healthcare) pre-equilibrated with 20 mM Tris-HCl, pH 8.0, 150 mM NaCl, 1 mM TCEP. The column was calibrated using gel filtration standard (Bio-Rad).

### SAXS measurement

SAXS measurements were performed at room temperature at the 12ID-B beamline of the Advanced Photon Source (APS), Argonne National Laboratory. A 14-KeV X-ray beam was used as the photon source with a sample-to-detector distance of 3 m for SAXS setup. Simultaneous wide-angle X-ray scattering (WAXS) data were recorded as well. Thirty two-dimensional (2D) images were recorded for each buffer and sample solution using a flow cell, with an exposure time of 0.5–1 seconds to minimize radiation damage. Concentration series measurements for the same sample were carried out to remove the scattering contribution due to inter-particle interactions and to extrapolate the data to infinite dilution. The concentrations were 0.75, 1 and 5 mg/ml for RC3H1(a.a. 1–445) and 1, 3, 5 and 10 mg/ml for RC3H2 (a.a. 1–442) in a final buffer of 20 mM Tris-HCl, pH 8.0, 150 mM NaCl, 1 mM TCEP. The 2D images were reduced to 1D scattering profiles using the MATLAB software package at the beamline. The 1D SAXS profiles were grouped by sample and averaged, after which the buffer background was subtracted. The procedures for data collection, processing, and analysis are similar to those previously described^50^. Low-resolution *ab initio* shape envelopes were determined using the program DAMMIN from the ATSAS package^51^. Thirty-two independent runs were performed, and the resulting models were averaged by DAMAVER, superimposed by SUPCOMB based on normalized spatial discrepancy (NSD) criteria, and filtered using DAMFILT to generate the final model.

## Additional Information

**How to cite this article**: Zhang, Q. *et al.* New Insights into the RNA Binding and E3 Ubiquitin Ligase Activities of Roquins. *Sci. Rep.* **5**, 15660; doi: 10.1038/srep15660 (2015).

## Supplementary Material

Supplementary Information

## Figures and Tables

**Table 1:** Data collection and refinement statistics.

	RC3H2(4Z30)	RC3H2/RNA(4Z31)	RC3H1(4YWQ)
Data collection			
Space group	P4_3_2_1_2	P2_1_	C2
Cell dimensions			
*a*, *b*, *c* (Å)	144.8, 144.8, 56.1	60.3,174.7,61.3	171.2,29.6,59.9
α, β, γ (°)	90.0, 90.0, 90.0	90.0,114.1,90.0	90.0,101.6,90.0
Resolution (Å)	50.0–2.71(2.76–2.71)	50.0–2.5(2.54–2.50)	50.0–1.70(1.73–1.70)
*R*_sym_ or *R*_merge_	0.083(0.905)	0.083(0.830)	0.125(0.729)
*I*/σ*I*	31.7(2.1)	25.4(2.4)	22.6(2.0)
Completeness (%)	100.0(99.9)	99.1(97.6)	99.6(99.9)
Redundancy	9.6(9.7)	5.9(5.4)	4.8(4.3)
Refinement			
Resolution (Å)	50.0–2.71	50.0–2.5	50.0–1.70
No. reflections	15846	38676	32036
*R*_work_/*R*_free_	0.220/0.253	24.5/25.9	19.9/23.7
No. atoms			
Protein	2237	4542	2483
RNA	0	1124	0
Water	13	40	184
*B*-factors			
Protein	83.3	62.6	21.9
Ligand/ion	n/a	74.5	31.5
Water	60.4	56.5	
R.m.s. deviations			
Bond lengths (Å)	0.010	0.007	0.009
Bond angles (°)	1.409	1.094	1.388
Ramachandran plot			
Favored regions (%)	98.0	98.8	99.1
Allowed regions (%)	2.0	1.2	0.9
Disallowed regions (%)	0.0	0.0	0.0

Values in parentheses are for the highest-resolution shell.
