# Supplementary material for: New Insights into the RNA-Binding and E3 Ubiquitin Ligase Activities of Roquins
Source: Sci Rep. 2015 Oct 22;5:15660. doi: 10.1038/srep15660 (PMC4614863; doi:10.1038/srep15660)
Supplement: Supplementary Information [file srep15660-s1.doc]

# New Insights into the RNA Binding and E3 Ubiquitin Ligase Activities of Roquins Authors: Qi Zhang^1^, Lixin Fan^2^, Feng Hou^1^, Aiping Dong^1^, Yun-Xing Wang^3^, and Yufeng Tong^1,4,*^

1. Structural Genomics Consortium, University of Toronto, Toronto, ON M5G 1L7, Canada
2. Small-Angle X-ray Scattering Core Facility, Center for Cancer Research of the National Cancer Institute, Frederick National Laboratory for Cancer Research, Leidos Biomedical Research, Inc., Frederick, MD 21702, USA
3. Protein-Nucleic Acid Interaction Section, Structural Biophysics Laboratory, National Cancer Institute at Frederick, National Institutes of Health, Frederick, MD 21702, USA
4. Department of Pharmacology and Toxicology, University of Toronto, Toronto, ON M5G 1L7, Canada

^*^Corresponding author: [yufeng.tong@utoronto.ca](mailto:yufeng.tong@utoronto.ca)

Supplementary Information

**Supplementary Figures**

**
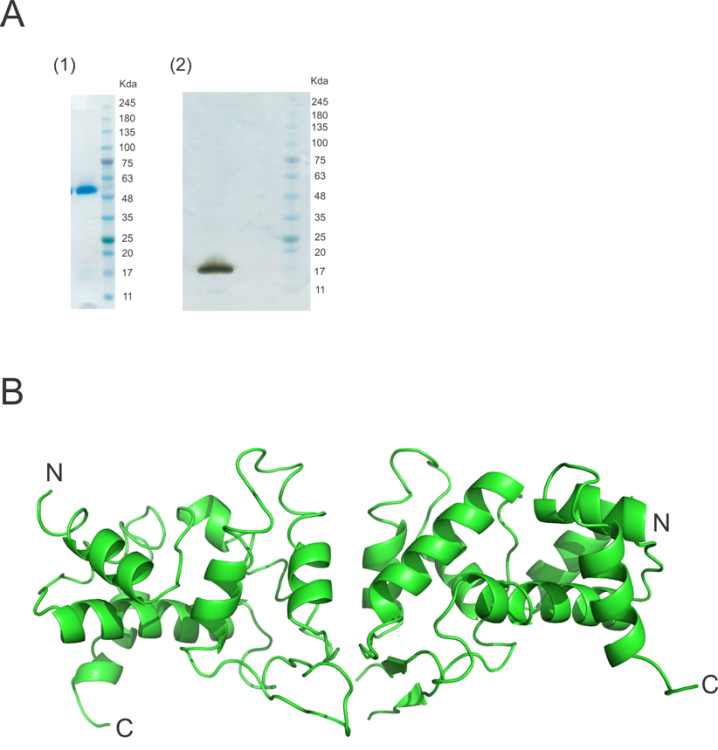
**

**Figure S1.** Crystallization and structure of RC3H1. (A) Crystallization of RC3H1. Left panel (1), the size of RC3H1 (a.a. 1–445) used to setup crystallization trial is about 50 kDa. Right panel (2), the polypeptide in the crystal has a size of about 17 kDa only, visualized by SilverQuest silver staining kit (Life Technologies). (B) The ROQ domain structure of RC3H1. ROQ forms a dimer in the ASU.

**
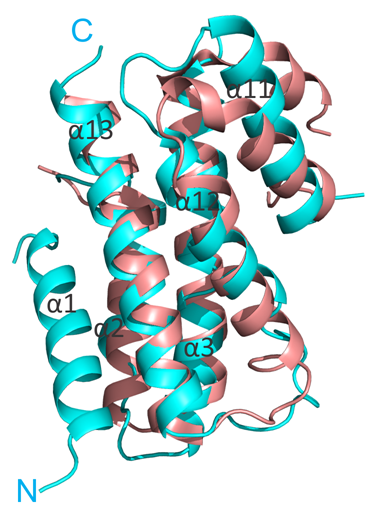
**

**Figure S2.**  Alignment of the HEPN domain of RC3H2 (cyan) with SsR10 protein from *Sulfolobus solfataricus* (PDB: 2Q00, brown).

**
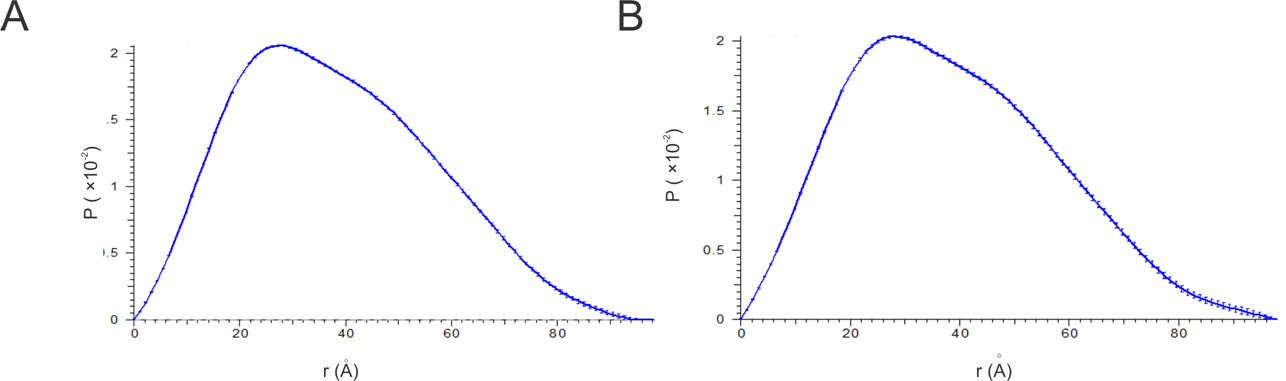
**

**Figure S3.** The pair-distance distribution function of both RC3H2 (A, a.a. 1–442) and RC3H1 (B, a.a. 1–445) showed an atypical bimodal distribution.

**
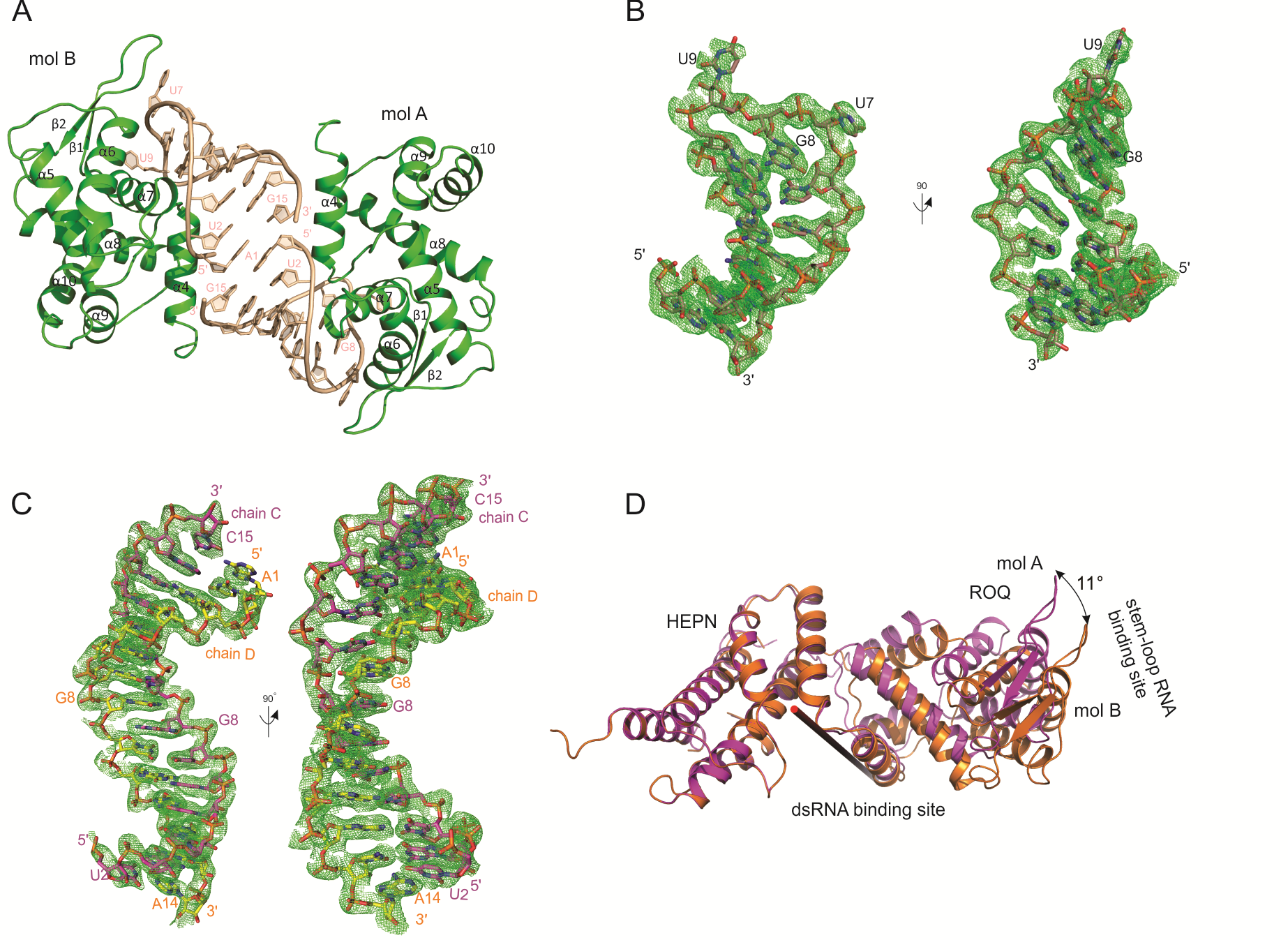
**

**Figure S4**. Interaction of RC3H2 with RNAs. (A) Stacking of stem-loop RNA nucleotide A1 with a second A1 from a symmetry related RNA molecule. (B) The omit *Fo-Fc* electron density map for the stem-loop form Ier3 RNA, contoured at 2.5 σ. Trinucleotide U_7_G_8_U_9_ are labelled. (C) The omit *Fo-Fc* electron density map for the Ier3 dsRNA, contoured at 2.5 σ, where nucleotides A1 in chain C and C15 in chain D are disordered and not visible in the map. (D) Comparison of the two RC3H2 protein molecules in the ASU of RC3H2/RNA complex. There is a 11° domain movement of the ROQ domains between the two molecules.

**
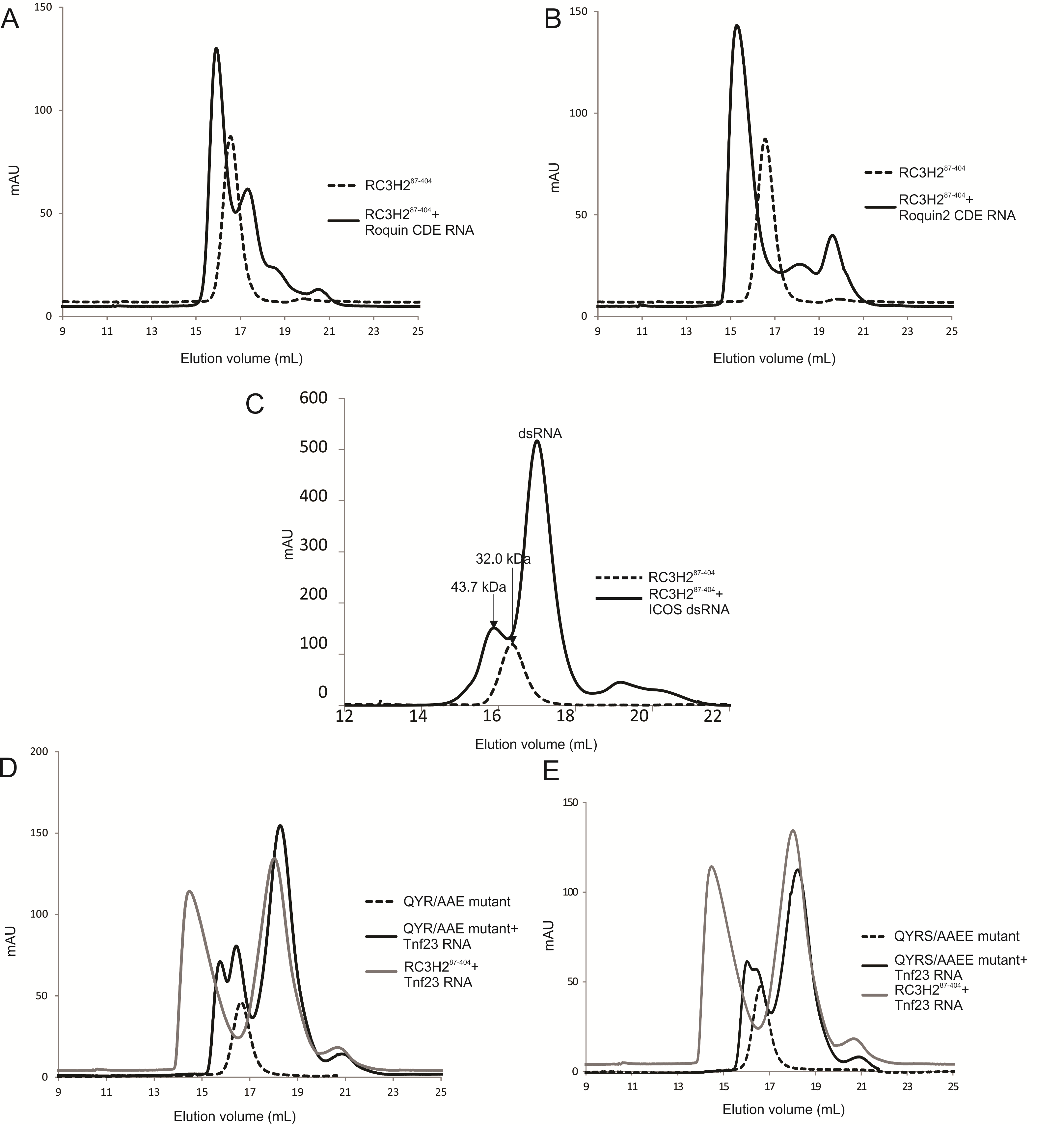
**

**Figure S5**. Analytical size-exclusion chromatography (SEC) analysis of the oligomeric state of the wild type and mutant RC3H2 (a.a. 87–404) in complex with different RNAs. (A) RC3H2 in the absence (dashed line) and presence (solid line) of 19-nt Roquin CDE RNA. (B) RC3H2 in the absence (dashed line) and presence (solid line) of 27-nt Roquin-2 CDE RNA. (C) RC3H2 in the absence (dashed line) and presence (solid line) of ICOS dsRNA. (D) Wild type (grey line) RC3H2 in complex with Tnf23 CDE RNA compared to stem-loop triple mutant RC3H2 in apo- (dashed line) and in Tnf23 CDE RNA binding forms (solid line). (E) Wild type (grey line) RC3H2 in complex with Tnf23 CDE RNA compared to phosphorylation-mimic plus stem-loop quaternary mutant RC3H2 in apo- (dashed line) and in Tnf23 CDE RNA binding forms (solid line).

**
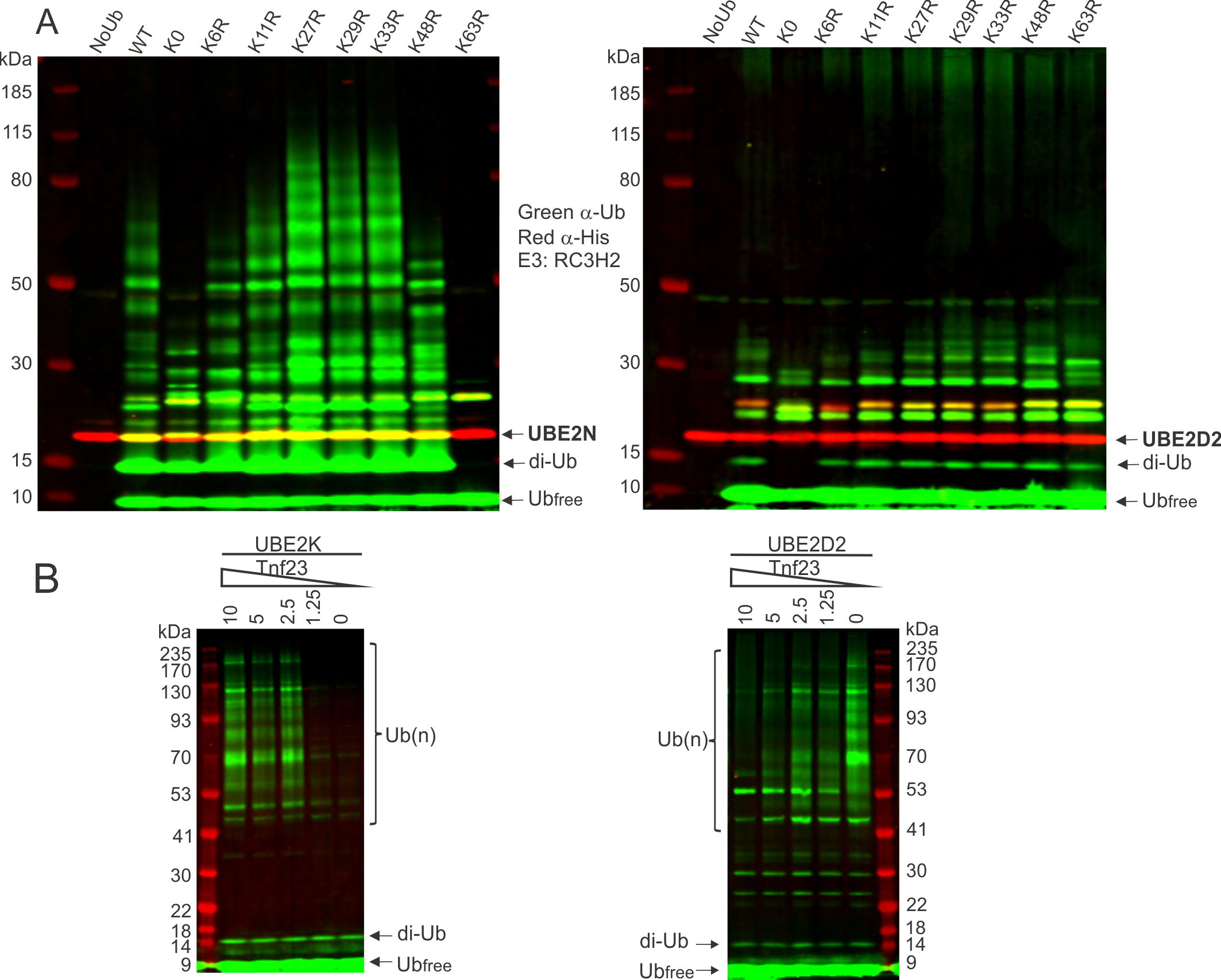
**

**Figure S6.** Ubiquitination and RNA binding of RC3H2. (A) Polyubiquitin chains of different linkage driven by RC3H2 when paired with UBE2N/UBE2V1 (left) and UBE2D2 (right). Each gel was double immunoblotted with anti-Ub (green) and anti-His (red) antibodies. UBE2N and UBE2D2 were His-tagged, while RC3H2 was not tagged. K0 stands for a ubiquitin mutant with all seven lysines mutated to arginine while other mutants have only one lysine mutated to arginine. (B) Effect of Tnf_23_ RNA on the auto-ubiquitination reaction by RC3H2 when paired with UBE2K (left) and UBE2D2 (right). Tnf23 RNA and RC3H2 were mixed at molarity ratios of 10:1, 5:1, 2.5:1, 1.25:1, and 0:1 before setting up the auto-ubiquitination assay. Each gel was only single immunoblotted with anti-Ub antibody. See *Methods* for more details of the assay.

**
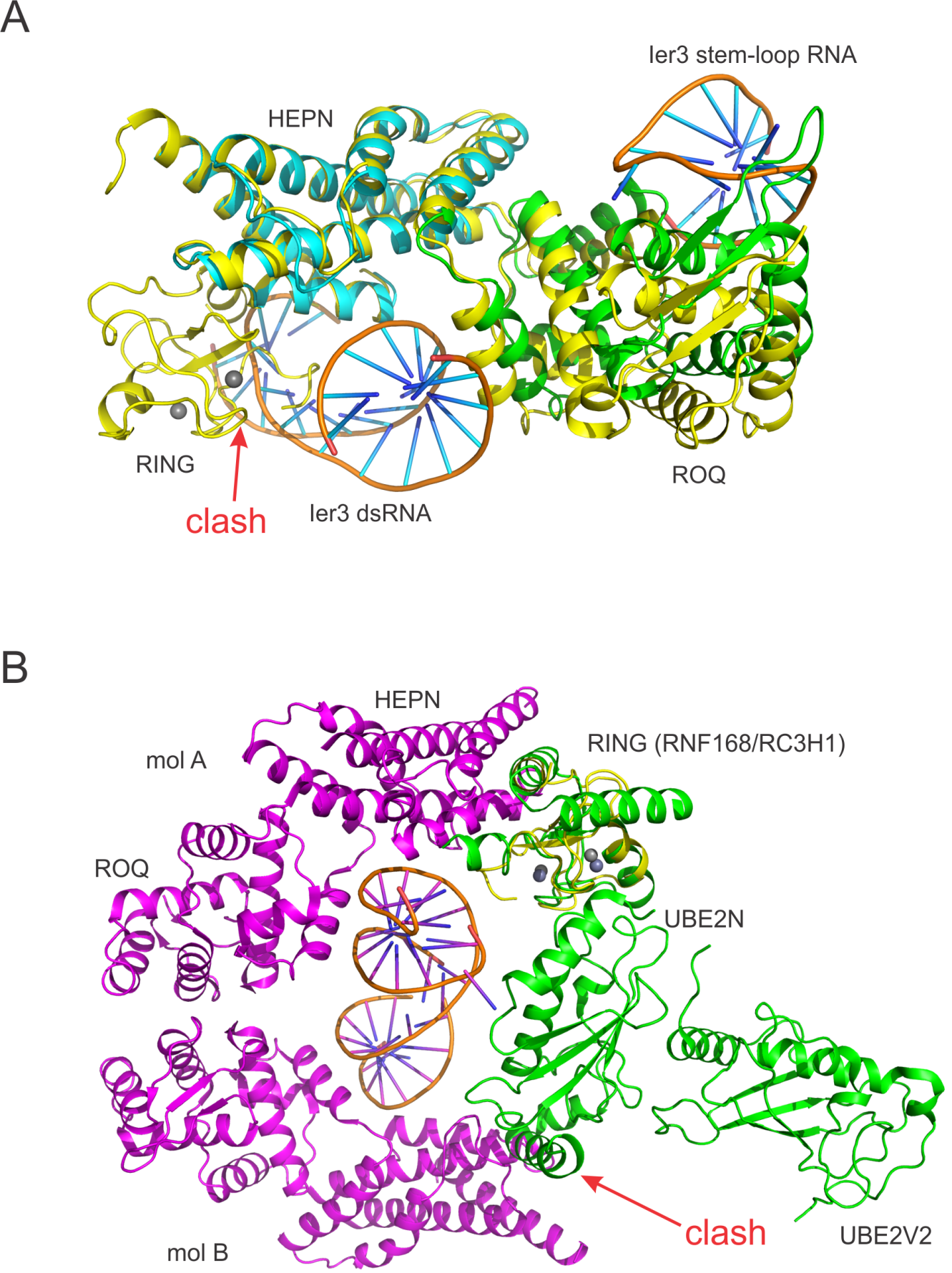
**

**Figure S7.** Modeling RNA binding and E2 interaction of Roquins. (A) Superposition of RC3H1 apo-form (yellow, PDB: 4TXA) with RC3H2-Ier3 complex structure in reference to the HEPN domain (ROQ domain in green and HEPN in cyan). Ier3 dsRNA is in clash with the RING domain. (B) Modelling of RING/UBE2N-UBE2V2 E2 complex (pdb:4ORH) onto the complex structure of RC3H1/Tnf23 dsRNA structure (PDB:4QIK). UBE2N is in clash with the second RC3H1 molecule that binds to the RNA duplex.

**
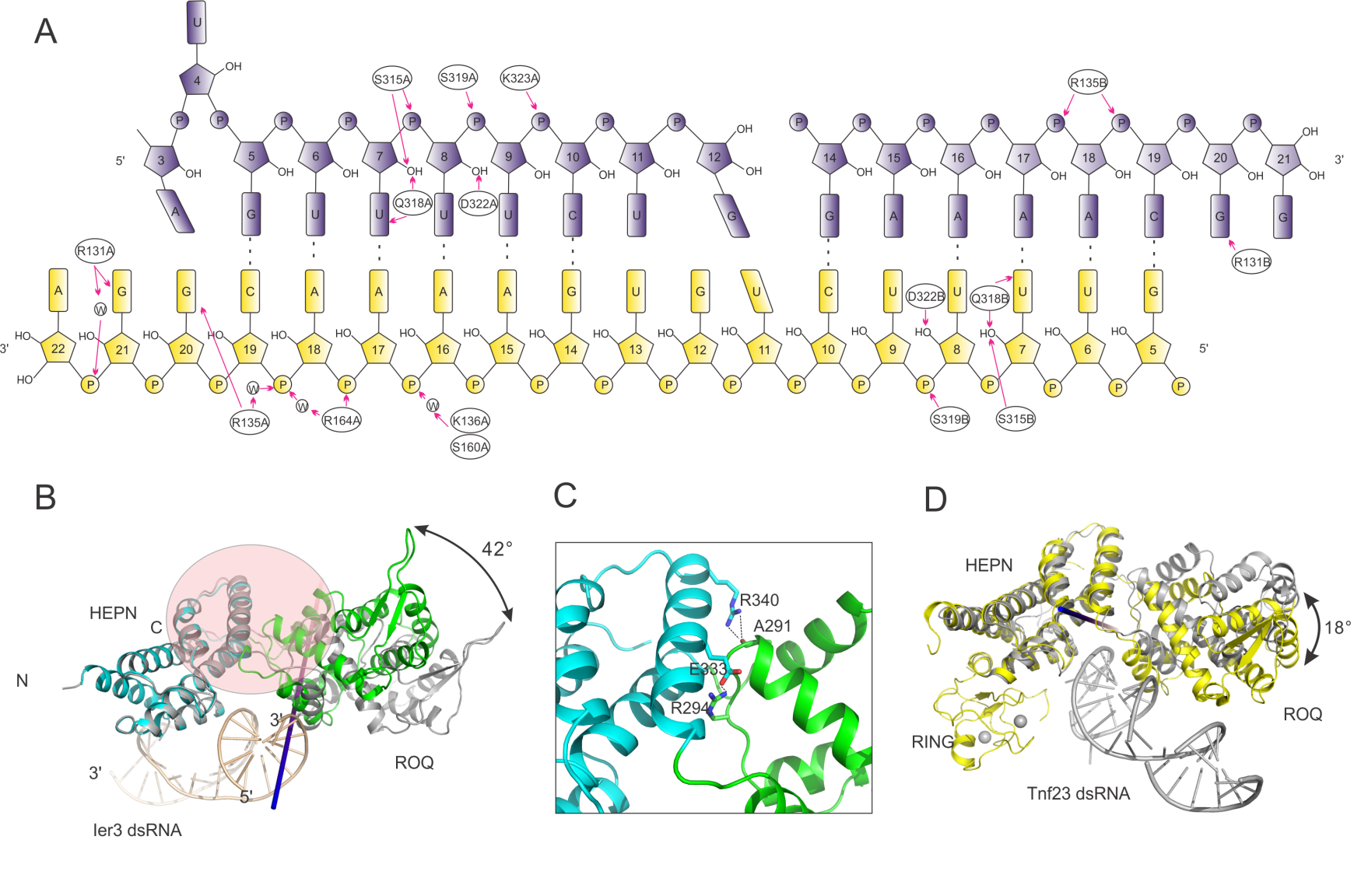
**

**Figure S8.** Comparison of different conformational states of Roquins.  (A) Schematic drawing of RC3H1-TNF _23_ dsRNA interaction. (B) Comparison of RC3H2 (a.a. 87-404) structures in apo- (grey) and RNA-bound forms (ROQ domain in green and HEPN domain in cyan). The stem-loop RNA is not shown for clarity. (C) A close up view of the interaction between the ROQ and HEPN domains upon dsRNA binding, i.e. the salmon coloured region in panel (B). (D) Comparison of RC3H1 HEPN/ROQ domain structures in apo- (yellow, PDB:4TXA) and Tnf23 RNA bound form (grey, PDB:4QIK). Cartoon representation prepared by Q.Z.


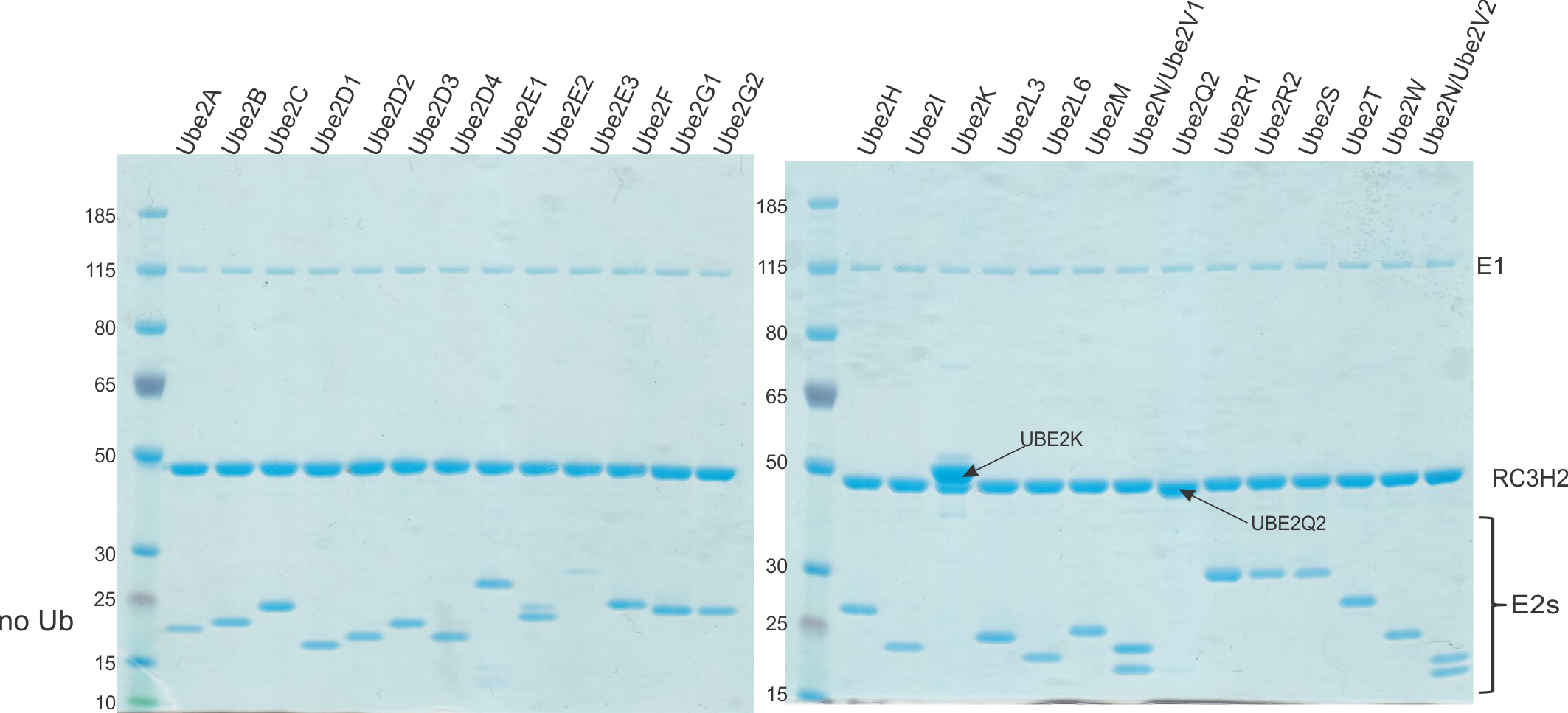


**Figure S9**. Negative control experiment of the auto-ubiquitination assay without adding ubiquitin. The same reaction systems were setup for all the E2 enzymes except that no ubiquitin was added. The gels were stained with Coomassie Brilliant Blue. Basically the gels demonstrated the amount of E1, E2, and E3 enzymes used in the reaction systems, and showed there were no contamination bands in the samples used.

**Supplementary Tables**

**Table S1.** Summary of the interactions between Roquins and different stem-loop RNAs.

| Ier3  RC3H2 | Phosphate group | nucleobase | ribose | Hmg19  RC3H1 | Phosphate group | nucleobase | ribose | Tnf23  RC3H1 | Phosphate group | base | ribose |
| --- | --- | --- | --- | --- | --- | --- | --- | --- | --- | --- | --- |
|  |  |  |  | C1 |  |  |  | A3 |  |  |  |
|  |  |  |  | U2 |  | W184  F194 |  | U4 |  | W184  F194 | R188 |
| A1 | R185 |  |  | C3 |  |  |  | G5 |  |  |  |
| U2 |  |  |  | C4 | R188 |  |  | U6 | R188 |  |  |
| G3 | S235 |  |  | C5 | S238 |  |  | U7 | S238 |  |  |
| U4 | T237 |  |  | U6 | T240 |  |  | U8 | T240  K239 |  |  |
| U5 |  |  |  | U7 | K239 |  |  | U9 | K239 |  |  |
| C6 |  |  |  | C8 | K220 |  |  | C10 | K220 |  |  |
| U7 | S261 | D260 | S262 | U9 | S264,  K220,R219 |  | S265 | U11 | S264 | D263 | S265 |
| G8 | R216  Y247 | R216  Q244 | Q244(OH) | G10 | R219  Y250 | R219  Q247 | Q247(OH) | G12 | R219  Y250 | R219  R247 | Q247(OH) |
| U9 |  | S250  R248 |  | U11 |  | S253  R251 |  | U13 |  | R251  S253 |  |
| G10 | R248 |  |  | G12 | R251 |  |  | G14 | R251 |  |  |
| A11 |  |  |  | A13 |  |  |  | A15 |  |  |  |
| A12 |  |  |  | A14 |  |  |  | A16 |  |  |  |
| C13 |  |  |  | G15 |  |  |  | A17 |  |  |  |
| A14 |  |  |  | G16 |  |  |  | A18 |  |  |  |
| C15 |  |  |  | G17 |  |  |  | C19 |  |  |  |
|  |  |  |  | G18 |  |  |  | G20 |  |  |  |
|  |  |  |  | A19 |  |  |  | G21 |  |  |  |

**Table S2.** Isothermal titration calorimetry (ITC) measurement of RC3H2 and RNA interactions.

| Protein | RNA | Kd(µM) | n | ΔH (kCal·mol^-1^) | ΔS (cal·mol^-1^·deg) |
| --- | --- | --- | --- | --- | --- |
| RC3H2 WT | Ier3 | 0.29±0.10 | 1.39±0.03 | -11.48±0.32 | -8.54 |
| RC3H2 WT | Ier3 | 0.36±0.09 | 1.18±0.02 | -15.58±0.39 | -22.80 |
| Q244A/Y247A/R248E | Ier3 | ND* | ND | ND | ND |
| RC3H2 WT | Tnf23 | 0.14±0.05 | 1.11±0.02 | -16.34±0.40 | -23.50 |
| Q244A/Y247A/R248E | Tnf23 | 0.36±0.16 | 0.87±0.03 | -4.00 | 16.10 |
| Q244A/Y247A/R248E/S323E | Tnf23 | 0.59±0.31 | 0.76±0.04 | -3.96 | 15.20 |

*ND, not detected.
